# Tibial Intramedullary Nailing by Suprapatellar Approach: Is It Quicker and Safer?

**DOI:** 10.7759/cureus.29915

**Published:** 2022-10-04

**Authors:** Ullas Jayaraju, R Rammohan, Fady Awad, Komalpreet Kaur, James Brock, Anil Singhal, Glenn Clewer

**Affiliations:** 1 Trauma and Orthopaedics, Prince Charles Hospital, Merthyr Tydfil, GBR

**Keywords:** radiological and clinical fracture healing, operating theatre, tibia shaft fracture, tibia nail, suprapatellar

## Abstract

Background

With the increasingly accepted method of suprapatellar tibial nailing for tibial shaft fractures, we aimed to compare intraoperative and postoperative outcomes of infrapatellar (IP) vs suprapatellar (SP) tibial nails.

Methods

This is a retrospective cohort analysis of 34 SP tibial nails over three years vs 24 IP tibial nails over a similar time frame. We compared total radiation dose (TRD), patient positioning time (PPT), fracture healing and follow up time. Knee pain in the SP group was evaluated utilising the Hospital for Special Surgery (HSS) Knee Injury and Osteoarthritis Outcome Score (KOOS).

Results

Fifty-eight patients with a mean age of 43 years were included. Mean intraoperative radiation dose for SP nails was 61.78 cGy (range: 11.60-156.01 cGy) vs 121.09 cGy (range: 58.01-18.03 cGy) for IP nails (p < 0.05). Mean PPT for SP nails was 10 minutes vs 18 minutes for IP nails (p < 0.05). All fractures united in the SP group vs one non-union in the IP group. Mean follow up was 5.5 months vs 11 months in the IP and SP groups, respectively. Mean KOOS was 7 (range: 0-22) at six months for the SP group.

Conclusion

The semi-extended position (SP group) leads to reduced TRD because of ease of imaging. Patients showed improved outcomes with shorter follow up and fracture union in all patients (SP group). The KOOS revealed that SP nail patients had minimal pain and good knee function. This study establishes a management and patient-reported outcome measures (PROMs) baseline for ongoing evaluation of SP nails.

## Introduction

Intramedullary nailing is a standardised treatment modality for the management of tibial diaphyseal fractures with satisfactory clinical and functional recovery [1]. A favoured and more traditional approach to the proximal tibial is through an infrapatellar (IP) portal, either by a transpatellar tendon split or retracting the tendon medially or laterally [2]. Proximal third diaphyseal tibial fractures can be difficult to treat through this approach as hyperflexion of the knee is required to gain an adequate entry point, which can lead to further displacement of the fracture [2]. Furthermore, anterior knee pain is also a recognized complication of this procedure affecting between 10% and 40% of patients [3]. The suprapatellar (SP) approach was developed to overcome these difficulties. The semi-extended position of the leg means the limb is adequately supported without the need for ongoing manual maintenance of reduction as well as simple positioning and use of the image intensifier, overall leading to a more efficient operation without compromising the outcome [4,5].

The SP approach is widely associated with reduced postoperative anterior knee pain, improved postoperative functional outcomes and an overall reduction in operative time [6]. Furthermore, suprapatellar tibial nailing is correlated with reduced intraoperative total radiation dose (TRD) compared to infrapatellar tibial nailing [6]. With the suprapatellar approach becoming more recognised and utilised in the management of tibial diaphyseal fractures, this study was performed to compare SP vs IP tibial nail fixations. The aim was to understand if SP tibial nail fixation had a reduced patient positioning time (PPT), with less intraoperative radiation and a reduced incidence of anterior knee pain than compared to IP tibial nail fixation. We will evaluate patient positioning time (PPT), total radiation dose (TRD) and anterior knee pain in suprapatellar tibial nailing via the Hospital for Special Surgery (HSS) Knee Injury and Osteoarthritis Outcome Score (KOOS).

## Materials and methods

A retrospective review of patients undergoing intramedullary tibial nailing for tibial diaphyseal fractures between January 1, 2015, and January 31, 2019. This cohort was divided into two groups based on the surgical approach used to access the proximal tibia entry point - suprapatellar group and infrapatellar group. Patient demographics, surgical approach, patient positioning time before commencement of operation and intraoperative TRD (measured in units of cGy) were collected for all patients from each group. Operative notes were reviewed to record intraoperative complications. Patients were followed up until discharge to assess postoperative complications, fracture healing and anterior knee pain.

The primary measure of outcome was patient positioning time and the secondary outcome measures were (1) suprapatellar TRD vs infrapatellar TRD and (2) fracture healing, by review of radiographs to assess for radiological evidence of healing. All the radiological images were independently reviewed by two trauma and orthopaedic consultants. We also wanted to evaluate anterior knee pain following suprapatellar tibial nail fixation through the KOOS questionnaire.

Statistical analysis was performed with the R statistics software version 3.5.1 (Vienna, Austria: R Foundation for Statistical Computing). Descriptive statistics were reported as the mean±standard deviation (SD), minimum-maximum, number and percentage. Mann-Whitney U test was performed for continuous non-parametric data and chi-square test for categorical variables to determine statistical significance. A p-value of <0.05 was considered to be statistically significant. The null hypothesis for this study was that there was no difference in the outcome measures between the two groups.

## Results

A total of 58 cases of tibial intramedullary nailing were included in this study. Of these patients, 24 cases were performed via an infrapatellar approach and 34 cases were performed using a suprapatellar approach. The demographics of patients included in the study are summarised in Table 1.

**Table 1:** Summary of the demographics of patients IP: infrapatellar; SP: suprapatellar

Demographic	IP group	SP group
Age at the time of procedure (years)	50.19 (range: 18-81)	33.0 (range: 17-66)
Follow up period (months)	6.5	11.27

The fractures were classified according to Arbeitsgemeinschaft für Osteosnthesefragen (AO)/Orthopaedic Trauma Association (OTA) classification system and the most common fracture pattern in both groups was the distal third level of tibial diaphysis (Table 2). The classification, indication for surgery, duration of follow up, union and radiation dose for the infrapatellar and suprapatellar groups are summarised in Tables 3, 4.

**Table 2:** Indications and fracture patterns IP: infrapatellar; SP: suprapatellar

Nature of injury	SP group	IP group
Open fractures	0	2
Closed fractures	32	22
Pathological fracture	1	0
Metastatic lesion	1	0

**Table 3:** Summary of data for infrapatellar group

Case	Age (years, at time of operation)	Level of fracture/lesion	Duration of follow up (months)	Radiation (cGy)	Time to position (minutes)	Union
1	20	Distal 1/3	6	85.99	8	Healed
2	43	Distal 1/3	10	140.2	31	Healed
3	28	Distal 1/3	12	139.19	16	Healed
4	18	Distal 1/3	13	116.28	23	Healed
5	22	Distal 1/3	11	94.84	14	Healed
6	22	Distal 1/3	7	155.6	33	Healed
7	24	Distal 1/3	7	102.31	31	Healed
8	40	Distal 1/3	32	96.09	27	Healed
9	22	Distal 1/3	6	97.21	12	Healed
10	44	Distal 1/3	6	89.84	12	Healed
11	57	Distal 1/3	7	74.21	9	Healed
12	37	Mid shaft	nil	146.12	5	Unknown
13	17	Mid shaft	8	272	28	Healed
14	32	Distal 1/3	6	131.8	22	Healed
15	36	Distal 1/3	2	81.35	15	Healed
16	66	Distal 1/3	6	63.53	18	Healed
17	27	Distal 1/3	12	58.01	14	Healed
18	21	Mid shaft	14	91.56	23	Healed
19	19	Distal 1/3	32	181.17	30	Non-union
20	33	Distal 1/3	nil	74.8	8	Not known
21	24	Distal 1/3	10	183.03	25	Healed
22	36	Proximal 1/3	27	155.24	12	Healed
23	35	Distal 1/3	5	95.9	0	Healed
24	56	Distal 1/3	9	179.9	0	Healed

**Table 4:** Summary of data for suprapatellar group

Case	Age (years, at time of operation)	Level of fracture/lesion	Duration of follow up (months)	Radiation (cGy)	Time to position (minutes)	Union
1	75	Mid shaft RCC mets no fracture	3	50.88	9	N/A
2	65	Distal 1/3	6	78.95	7	Healed
3	32	Mid shaft	17	75.42	14	Healed
4	53	Distal 1/3	11	82	15	Healed
5	56	Distal 1/3	0	94.06	11	Lost to follow up
6	78	Distal 1/3	0	69.65	10	Lost to follow up
7	69	Distal 1/3	13	63.9	11	Healed
8	38	Mid shaft	5	58.49	5	Healed
9	51	Distal 1/3	7	156.09	8	Healed
10	71	Proximal 1/3	5	105.2	11	Healed
11	50	Distal 1/3	8	112.29	12	Healed
12	25	Mid shaft	8	122.56	11	Healed
13	40	Distal 1/3	6	83.07	13	Healed
14	49	Mid shaft	4	151.05	14	Healed
15	78	Proximal 1/3	4	68.01	7	Healed
16	72	Distal 1/3	4	109.49	17	Healed
17	18	Distal 1/3	4	77.91	10	Healed
18	55	Distal 1/3	3	74.7	14	Healed
19	24	Distal 1/3	0	106.7	12	Healed
20	72	Mid shaft	0	78.15	6	Healed
21	47	Distal 1/3	6	44.9	11	Healed
22	39	Distal 1/3	4	14.15	10	Healed
23	21	Distal 1/3	7	17.06	6	Healed
24	22	Distal 1/3	6	20.07	9	Healed
25	71	Distal 1/3	3	21.58	11	Healed
26	54	Distal 1/3	9	18.6	10	Healed
27	71	Distal 1/3	0	29.2	8	Lost to follow up
28	33	Mid shaft	0	28.9	7	Healed
29	21	Mid shaft	0	19.76	9	Healed
30	50	Distal 1/3	0	14.8	7	Healed
31	34	Distal 1/3	0	11.78	8	Healed
32	81	Mid shaft	0	11.6	10	Healed
33	54	Mid shaft	0	11.57	12	Healed
34	40	Mid shaft	0	17.9	12	Lost to follow up

The most common indication for surgery was acute fracture following trauma and there were no wound-related complications, deep infections, compartment syndrome or deep vein thrombosis/pulmonary embolism reported in either group. The mean follow up for the suprapatellar group was 6.5 months and for the infrapatellar group was 11.3 months. Twenty-one patients in the suprapatellar group went on to achieve full union, one patient developed non-union and two were lost to follow up. Twenty-one patients in the suprapatellar group went on to achieve full union, one patient had the nail for metastatic renal cell carcinoma and therefore fracture union was not an applicable outcome measure and 12 patients were lost to follow up. The primary outcome measures are summarised in Table 5.

**Table 5:** Primary measures of outcome IP: infrapatellar; SP: suprapatellar

Outcome measures	IP group	SP group	p-Value
Time taken for positioning (minutes)	18.9 (SD: 8.496, range: 5-33)	10.2 (SD: 2.76, range: 5-15)	< 0.05
Total Intraoperative fluoroscopic radiation (cGy)	121.09 (SD: 48.6, range: 58.01-183.03)	61.8 (SD: 41.2, range: 11.57-156.09)	< 0.05

The mean PPT in the suprapatellar group was 10.2 minutes compared to 18.9 minutes in the infrapatellar group (p = 0.0001). The TRD in the suprapatellar group was 61.8 cGy compared to 121.09 cGy in the infrapatellar group (p < 0.00001). The mean KOOS score for the SP group was 7.34 and the difference in healing rates was not statistically significant (chi-square test with continuity correction, p = 1).

There was one case of non-union in the infrapatellar group. Further evaluation of this case revealed a 20-year-old male smoker who presented with an open fracture (Gustilo-Anderson classification grade 1 at the time of surgery) and went on to have an IP tibial nailing with primary closure. He underwent two further operations six weeks after the primary operation and definitive fixation for a washout of an infected haematoma and removal of a distal locking screw. At 14 months postoperatively, due to persistent pain and lack of radiographic union, the patient underwent computed tomography (CT) scan which confirmed a fracture non-union. He was commenced on EXOGEN therapy (ultrasound bone healing system for long bone fractures with non-union or delayed healing) for 6 months but this did not improve clinical outcomes. He is currently awaiting revision surgery.

## Discussion

In the current study, we were able to demonstrate shorter patient positioning time and lower intraoperative TRD for tibial intramedullary nailing utilising the suprapatellar approach.

The semi-extended position of the knee in the suprapatellar approach allows for quicker PPT and is not demanding in maintaining fracture reduction/position. In comparison, the infrapatellar approach requires at least 90° of knee flexion or hyperflexion to introduce the entry guide-wire and subsequent tibial nail [7]. Various techniques are adopted by surgeons to maintain the flexed position of the knee, which involve additional attachments, supports and manual fracture reduction, optimal patient positioning for the infrapatellar approach can be time-consuming [7]. Whereas the semi-extended position required for suprapatellar approach is less demanding with a simple set-up [8]. The semi-extended position also facilitates maintenance of fracture reduction and reduces the risk of malalignment [9,10]. There is minimal evidence in literature comparing patient positioning time in IP tibial nailing vs SP tibial nailing. We have shown that there is shorter PPT in SP tibial nail fixations, due to the simple set required for the semi-extended position. This can, potentially, impact a surgical case by reducing operation and anaesthetic times. PPT and the semi-extended position have multiple further advantages in SP tibial nailing, by allowing easier access and optimal intraoperative radiographs via the image intensifier, without the need for intricate image intensifier positioning [10]. In addition, during the IP approach the absorbed TRD is higher (as outlined in Tables 3, 4), this could be a result of x-rays entering obliquely, increasing the cross-section of exposure [10,11]. Another positive impact of the semi-extended position in SP tibial nailing is reduction in TRD compared to the IP group. A number of studies found similar results when comparing SP tibial nailing vs IP tibial nailing, with the conclusion that SP tibial nailing had reduced operative times and TRD compared to the IP tibial nailing [11,12].

We did not observe a statistically significant difference in fracture healing in our study. A prospective randomised control pilot study between SP and IP approach for tibial nailing by Chan et al. also found no difference in fracture healing. As well as in this study, they did not find any difference in anterior knee pain, functional disability, or knee range of motion [13]. Similar results were noted by Gao et al., in their meta-analysis of randomised control trials comparing the two approaches [13].

Anterior knee pain and damage to patellofemoral joint (PFJ) cartilage are known complications of IP and SP approaches, respectively [14]. The available evidence appears equivocal with some authors demonstrating no patellofemoral articular cartilage damage while some cadaveric studies show damage to patellofemoral in one-third of study specimens, but the long-term clinical outcomes of this are yet unclear [15]. It was beyond the scope of this study to review this complication specifically. However, we did review postoperative anterior knee pain in the SP group, which is a well-established postoperative complication of IP tibial nailing. Published literature appears to show similar incidences of anterior knee pain in both infrapatellar and suprapatellar groups with some studies reporting reduced anterior knee pain in SP group [16,17]. We utilised the KOOS patient-reported outcome measures (PROMs) questionnaire for calculating knee pain following SP tibial fixation. For the SP group, we found a mean score of 7, which reveals a low pain score for the SP group. In this study, we did not review postoperative anterior knee pain for the IP group, therefore, we can definitively compare knee pain between the two groups. However, we can state that our findings for anterior knee pain following SP tibial nailing are in keeping with current literature [16-18].

From our study, we have been able to establish a shorter patient positioning time, with potentially shorter operative time and reduced TRD for SP tibial nail fixations. Several large studies including systematic reviews and meta-analyses demonstrate the superiority of the SP approach for tibial intramedullary nailing in comparison to the IP approach, an opinion we indeed share and corroborate with, in this study [18-20]. However, these studies have not evaluated PPT, which we believe is an important aspect of tibial nailing, and one which can reduce overall operative and anaesthetic times and the semi-extended position can aid in a reduction in TRD.

There are limitations to this study. We understand that this is a small heterogeneous group of patients. Patients lost to follow up and unmatched case-control groups are further limitations. In particular, there were 12 patients lost to follow up in the SP group.

## Conclusions

The suprapatellar approach for tibial intramedullary nailing provides a simple method for patient positioning, thereby reducing patient positioning time and subsequently helping reduce TRD compared to infrapatellar tibial nailing. With regards to outcomes of suprapatellar tibial nailing, we found all fractures healed and the KOOS PROMs highlighted a low postoperative pain score. We believe our study has shown suprapatellar nailing has multiple advantages, and we have established a PROMs baseline for the management of tibial nailing, and this should be continued to further evaluate this surgical technique.
